# Promoters and inhibitors of treatment adherence among HIV/AIDS patients receiving antiretroviral therapy in Ghana: Narratives from an underserved population

**DOI:** 10.1371/journal.pone.0230159

**Published:** 2020-03-06

**Authors:** Gladys Dzansi, Eric Tornu, Jeniffer Chipps

**Affiliations:** 1 Department of Adult Health, School of Nursing and Midwifery, College of Health Sciences, University of Ghana, Legon, Accra, Ghana; 2 School of Nursing and Midwifery, University of the Western Cape, Cape Town, South Africa; University of South Florida, UNITED STATES

## Abstract

Adherence to antiretroviral therapy (ART) is essential to achieving an improved cluster of differentiation-4 (CD4) count, viral load, and quality of life while preventing drug resistance. Medication adherence among people living with Human Immunodeficiency Virus (HIV) is influenced by different factors. Several studies have identified adherence promoters and inhibitors that emanate from the internal and external environment. We conducted six (6) individual in-depth interviews and three (3) focus group discussions to explore adherence behaviour among patients diagnosed with HIV in a teaching hospital in Accra, Ghana. Participants were drawn from the intervention arm of a mobile phone adherence intervention program. They had been enrolled in the study for at least six (6) months before the interviews are conducted. Results revealed that participants adhered to treatment irrespective of prompts from significant others. Adherence promoters included perception of ART as part of daily routines, benefits of the ART, awareness of regimen, access to food, and transparency. Adherence inhibitors were forgetfulness, secrecy, waiting time, religious beliefs, and sleep. People living with HIV (PLWHIV) have the personal motivation to take medication; however, negative perceptions about HIV must be addressed to ensure optimum adherence behaviour.

## Introduction

The Joint United Nations Programme on Human Immunodeficiency Virus (HIV)/Acquired Immune Deficiency Syndrome (AIDS) (UNAIDS) and World Health Organization (WHO) projected that about 36.9 million people were living with HIV, while 1.8 million were newly diagnosed as of 2017 [1,2]. Globally, HIV mortality has declined from 1.5 million in 2000 to 0.9 million in 2017, while treatment access and coverage have increased (2 million in 2000 to 21.7 million in 2017) with an estimated 59% of patients receiving treatment. However, the debilitating effect of HIV is still evident in Africa. About 53% of all persons living with HIV reside in Sub-Saharan Africa. Despite these high incidences, treatment coverage and access (15,358,000; 60%) have contributed to mortality declining by 35% compared with the year 2000 estimates.

The adult prevalence rate of HIV in Ghana in 2018 was 1.7%, with 20,000 new cases and 14,000 deaths recorded [2]. A notably low treatment access rate of 40% was recorded relative to global and regional averages. In the face of these gaps in access to HIV treatment, the importance of adherence behaviour in ensuring improvement in outcomes cannot be dismissed. Consequently, adherence monitoring is included as one of the pillars within the treatment modalities of HIV care.

Adherence to antiretroviral drugs (ARVs) is a major facilitator for improving the outcome of care for people living with HIV (PLWHIV). It promotes treatment effectiveness and minimises adherence-related drug resistance [1]. The consequence of non-adherence behaviour is associated with ineffective treatment, delayed remission of illness, drug resistance, prolonged or recurrent hospitalization, increased treatment cost, and a higher risk of death [3]. Furthermore, PLWHIV who have high viral loads and do not adhere to treatment are more likely to infect others, which increases HIV incidence rates and burden of care on the health systems. Despite the fact that optimum antiretroviral therapy (ART) adherence for some antiretroviral treatment is attained at 95% level of adherence [3], adherence behaviour levels, its outcome, and associated meaning may vary in different populations globally. Ortego, Huedo-Medina [4] in a meta-analysis compared the levels of adherence between male and female respondents and observed a 90% level of adherence in about 62% of patients receiving treatment. Levels of adherence among patients in parts of Ghana were also estimated to span from 73.6% to 91% [5].

Initiating ART, sustaining the treatment, and dealing with the associated biopsychosocial outcomes remains a challenge. Measures were introduced to reduce medication sideeffects, pills burden, and the dosing frequency, were identified as barriers to medication adherence. However, issues relating to the decision-making process in treatment adherence still persist.

Factors influencing adherence manifest as either promoters or inhibitors of adherence behaviour. Previous studies have suggested that PLWHIV are motivated to take treatment depending on the premium they place on the treatment, the benefits, self-efficacy, acceptance of diagnosis, and disclosure of status [6–9].

Adherence behaviour is linked to the importance of the medication and the benefits derived from taking the ART. Patients who experience an improvement in their health status as a result of medical care and adherence to treatment may become motivated to take medication voluntarily [10]. Starks et al. [9] conducted an in-depth study with 29 participants in Beijing and observed that patients’ will to live promoted adherence behaviour. Similarly, van Loggerenberg [10] mentioned that ART was perceived as a lifesaver among most patients in South Africa, hence, encouraged adherence behaviour.

Adherence is also better among patients with the knowledge, skills, and attitude that ensures consistency with medication schedules [11]. The adherence counselling session empowers patients to be conscious of the relevance of the ART and the long-term obligation [12]. Chung et al. [12] implemented adherence counselling sessions lasting at least 30 minutes. Trained counsellors in Kenya provided two (2) counselling sessions to patients before initiating treatment and a third session a month after treatment was started. They evaluated pharmacy replenishment records and patients’ virology status in the study. The outcome supported the effectiveness of counselling in promoting adherence behaviour, which contributed to the positive care outcomes for the patients. They further noted non-compliance with intention-to-treat analysis—a technique used in randomised control trials as a limitation. An in-depth report would have accounted for what led to the success of the counselling sessions. Nonetheless, patients’ adherent behaviour depends on the response to diagnosis and willingness to disclose status.

Patients’ acceptance of diagnosis and willingness to disclose status enhances social support, thereby facilitating adherence. Accepting diagnosis ensures prompt initiation of treatment, while denial leads to delay and poor health outcomes [11]. Portelli [11] affirmed this stance and noted that HIV status disclosure promoted support from relations, companions, and significant others. The contrary case is that fear of stigma leads to the patient’s failure to disclose HIV positive status, which results in inadequate support from their significant others [11]. Literature suggests that patients need support to remember their medication schedule and appointments but this would be feasible only when information about their status is disclosed.

Forgetting to take pills has been reported widely as a major barrier to treatment adherence in patients receiving ART [11,13]. Other patient-related factors contributing to non-adherence to ART include depression, stigma [7,11,14], lack of self-worth, age, low health literacy levels, preference for alternative treatment, denial of HIV status, limited financial resources, and cognitive impairment [7].

In view of the seeming contextual differences, we posed the following questions: What is the adherence behaviour among PLWHIV in Ghana? What factors support positive adherence outcomes? Why do some patients stop or miss taking their medication?

## Materials and methods

This was a phenomenological qualitative study. We conducted six (6) in-depth interviews and three (3) focus groups (20 participants) as part of Phase Three (3) of an intervention study that implemented an integrated mobile phone intervention. Participants in the intervention group of the two-arm randomized control trial were supported to set mobile phone alarms on medication time. Additionally, they received weekly text messages and monthly voice calls. Participants were assessed at baseline, then again at three and six months. Participants in the intervention group were purposively selected for in-depth interviews and focus groups in the sixth month. This enabled the researchers to elicit their experiences prior to the intervention and their evaluation of the integrated mobile phone intervention.

Adult participants (between 18–65 years of age) who were receiving ART in a teaching hospital in Accra, Ghana were recruited from February 2015 to April 2015 using purposive sampling. Thirty (30) out of 181 participants who met the inclusion criteria were invited to take part in the interview and focus group discussions. Twenty-six (26) accepted and were interviewed. The remaining four (4) participants were not available to be interviewed. The sample size for the interview was determined by data saturation–which is the point at which no new or additional ideas were being identified in the interviews. Participation included: use of alarms for medication adherence, receipt of weekly text messages and monthly voice calls for the six (6) months duration of the intervention. Participants in the intervention group who did not set alarms to monitor their medication or receive weekly messages and monthly voice calls were excluded from the interview phase.

The principal investigator (GD) and three (3) trained research assistants conducted the interviews with interview guides through face-to-face interaction with participants. Only the research assistants were present during individual interviews and focus group discussions. The interviewers’ familiarity and prior engagement with the participants facilitated an open discussion of sensitive issues that would not have been discussed if there was no relationship. The research assistants’ initial interviews were done with the principal investigator following the training until the principal investigator was satisfied with the interviewing skill of the volunteer. Satisfaction with the volunteer’s interviewing skills was based on the ability to take participants’ informed consent correctly, pose questions on the interview guide, and probe participants’ responses. Interviews were scheduled to coincide with participants’ regular clinic visits. A private room in the hospital was secured for the interviews. Participants’ written informed consent was sought for the interview, audio recording, and use of data. Code names were assigned to each participant to protect their privacy on the audio recordings. Questions and probes on the interview guide were used relative to the responses of the participants and study objectives.

Additionally, field notes were taken to guide the interpretation of data. Each interview lasted for about 30 minutes while the focus group discussions lasted for at least 60 minutes. The qualitative data collection lasted for a period of three (3) months (February to April 2015). No repeat interviews were conducted. We were unable to register the trial at the time of commencement; although, the protocol was submitted due to funding requirement. There was, however, rigorous review and monitoring by a three (3) member steering committee who managed the entire process. Written ethical clearance was obtained from the Noguchi Memorial Institute for Medical Research Institutional Review Board (NMIMR-IRB) (Approval Number: 039/13-14). With the aid of the study’s information sheet, the interviewers explained the study’s purpose, duration, and participants’ roles to them. The right to enroll, decline, or withdraw from the study was also explained and enforced. Participants received incentives in the form of transportation, mobile phone call credits, and snacks. This study adheres to the Consolidated Criteria for Reporting Qualitative Studies (COREQ) [15].

### Data analysis

We used Tesch’s [16] comprehensive guidelines for transcribing and analysing in-depth interview data, as reported by Creswell [17]. This involves describing, classifying, and connecting the elements of the data. The eight (8) specific steps were: transcription, extracting codes, clustering data, categorising, exhaustive description of data, category labelling, category, grouping category and description. We transcribed audio recordings verbatim and read the transcripts several times to make meaning of data. Each interview and focus group discussions transcript were uploaded into ATLAS Ti 7 software as separate files for coding. The codes were discussed among team members (*n* = 3); then clustering, categorising, and grouping of the code into themes and subthemes was completed. The themes and subthemes generated from the data were described in detail with direct quotes supporting the various categories. Although the interviews and focus groups were analysed separately, the data were discussed and integrated into the study findings. The focus groups were done to validate and provide additional information. Field notes were integrated into the findings to reflect the context of the narratives. No transcripts were returned to participants for comments or correction but clarity was sought during focus group discussions on aspects requiring more probing. The participants verified the findings verbally.

We ensured that data were credible by using various approaches in data collection, such as focus group discussions, face-to-face interviews, field observations, and document reviews. Although the interviewers had experiences in HIV care, biases were limited through reflexivity. The findings were verified through member checking. The findings have been reported with relevant details to facilitate transferability without compromising the privacy and confidentiality of participants. Participation was voluntary and participants’ privacy was ensured in line with the tenets of research ethics.

## Results

ART is a long-term treatment and requires lifetime commitment and consistency. Individuals taking ART may be either adherent or non-adherent to treatment. Participants reported their motivation for adhering to treatment and circumstances under which non-adherence occurred. Adherence behaviour from the participants’ perspective was influenced mostly by advice on diet, substance abuse, and meeting follow-up visit schedules.

The two (2) main themes derived were adherence promoters and adherence inhibitors. The subthemes for adherence promoters were: adherence to antiretroviral therapy as a routine, familiarity with medication regimen, benefits of ART, access to food, fear of death, pill mobility, status disclosure and voice calls. The subthemes for adherence inhibitors were: forgetfulness, religious beliefs, nondisclosure (secrecy), waiting time, and sleeping. The initials (FDG) were used to represent data extracts from focus group interviews while (P) represented individual interviews. Quotations from focus groups 1, 2 and 3 were designated as (FDG 1), (FDG 2) and (FDG 3), respectively.

The participants’ demographic characteristics are summarized in Table 1.

**Table 1:** Socio-demographic characteristics of study participants.

	Individual Interviews	Focus Group 1	Focus Group 2	Focus Group 3	Total
**Age** *Mean (SD)*	37(7.52)	41(1.86)	38(6.85)	47(5.81)	41(7.28)
**Gender**					
*Male*	3	2	6	5	14
*Female*	3	4	2	1	14
**Education**					
*Tertiary*	1	1	0	0	4
*Below tertiary*	4	0	2	2	8
*No education*	1	5	6	4	16
**Relationship**					
*Married*	5	6	4	4	21
*Not married*	1	0	4	2	7
**Children**					
*None*	2	0	5	0	7
*2 or less*	4	5	2	3	
*3 or more*	0	1	1	3	5
**Duration of illness**					
*Mean (SD)*	3(1.72)	4(2.14)	2(2.27)	5(2.58)	6(2.58)
**Treatment duration**					
*Mean (SD)*	2(1.72)	3(1.86)	2(1.69)	4(1.72)	5(1.85)
**Total participants**	**6**	**6**	**8**	**6**	**26**

### Adherence behaviour promoters

Participants acknowledged that they adhered to treatment as a routine and because of the benefits derived. They also noted that food played an important role in adhering to treatment since it reduced the undesired side effects of ARVs, such as weakness and dizziness. The participants mentioned that fear of death heightened the willingness to strictly follow the treatment regimen. Other participants indicated that they carried their pills with them to avoid missing or delaying medication time. In evaluating the intervention, voice calls were the preferred adherence support measure.

#### Adherence to antiretroviral therapy as a routine

Some participants revealed that strictly following their medication regimen was part of their daily routine. This was because they had been on the ARV treatment for over five (5) years; thus, taking the medication had become a well-established routine. More so, participants appreciated the consequences of non-adherence, such as treatment failure, and poor health status, which therefore challenged them to adhere to their medication regimen.

*As for the medicine*, *when you take it for some time you become used to it such that you do not need anyone to remind you to take it…I have followed exactly as I was taught during the counselling sessions to the extent that I have to run for my drugs when the time passes by a second*. *I always want to take the drugs on time so that I will be strong at all times…*.*during counselling we were informed and cautioned about the need to stick to the medication as prescribed and I have observed that if you follow the instructions about taking the medication then you have no problem*. (FDG 2)

The participants also revealed that they attended adherence counselling sessions at the clinic prior to initiating the treatment. They suggested that a minimum of two (2) counselling sessions with a trained adherence counsellor enhanced their knowledge about the medication and the relevant instructions to follow. It was also mandatory to attend the counselling sessions with an adherence monitor (family member or friend) who would ensure that support for adherence was provided.

#### Familiarity with medication regimen

Although participants were unable to mention the name of the medications, they had no problem identifying the medication and how these were to be taken. Most participants used the colour, shape and size, of medication to follow their medication regimen. Participants’ regimen was mostly once daily (QD) or twice daily (BID); and they had a pill or two to take in the morning, evening, or both morning and evening. There were no complaints of pill burden–that is, the high number of pills taken on a regular basis, which could deter participants from taking their medication.

Some of the participants acknowledged that the dosing regimen was changed during the course of treatment; but this did not affect their medication adherence. The side effects of drug and type of brand in stock necessitated the regimen changes; however, adherence to treatment was not affected by these modifications.

*Initially*, *some were twice daily dosing and once daily*. *Later it was changed again*. *Currently*, *the medication I have I take 2 in the morning and 1 at night*. (P1)

Field information revealed there were diverse brands of the ARVs. While some of the ARVs were combined to a single dose, others were not; and therefore, the number of pills and dosing varied. No matter the change in medication, participants were cognizant of the benefits they derived from taking the ARV’s and persisted in taking their medications.

#### Benefits of ART as a reason for adherence

The motivation for adherence was affected by the benefits of ART. Some participants, after comparing their present state of health with their past (prior to being put on ARVs), remarked that the medication was helpful. They described the medication as a “source of energy” for them to be able to go about their daily activities. Some acknowledged the action of the ARV as being able to fight the activities of the virus, thereby improving their general health.

*Just as my colleague mentioned*, *the medication that we take reduces the activity of the virus and gives us energy*. *Since I started taking the medication*, *I feel healthier and there is a difference in my energy levels*. *I feel very strong*. *I take my medication religiously because I know how taking it has helped me*. (FGD 2)

Participants recognised that taking the medication consistently was a catalyst for maintaining good health while living with HIV.

#### Access to food promotes adherence

Some participants suggested that access to food promoted their adherence to the ARVs. They mentioned experiencing undesired side effects of the ARVs (weakness and dizziness) when they took their medication without food. Participants added that eating a balanced diet also contributed to their recovery. A participant noted:

*The part about eating well before taking the medication is not a joke*. *The medication needs food*. *In the evening if you take the drug after eating*, *then it is able to work well*. *But if you haven’t eaten before taking it*, *then you will be in serious trouble*. *You might feel like someone who took alcohol or ‘wee’ [Marijuana]*. (FGD 1)

Participants of the focus group discussions mentioned that eating well before taking the medication was very important. Some noted that failure to eat before taking the medication worsened the side effects of the treatment. Access to food and eating the right combination of food, in their view, contributed to minimising treatment side effects, such as weakness, and ensured that their treatment regimen was not stopped or skipped. One participant argued that failing to eat before taking the medication could result in death.

#### Fear of death motivates adherence

Due to previous observations of how others died, three (3) participant opined that the fear of dying was a strong motivator for adherence to treatment. Recounting the events characterising the diagnosis of HIV, some of the participants appreciated that adhering to treatment had improved their health. Failure to adhere to treatment was perceived as a risk for poor health and death; hence, participants strictly adhered to the treatment regimen.

…*if it is time for the medication*, *and you don’t take your medication the disease will recur and if that happens it can kill you…Because you will be there and the illness will come back and if you are not careful you will die and leave your children…*(P3)

*I think the way I felt and was seriously sick I was afraid that I will die so it gave me the motivation to be more conscious about my medication*. (P1)

One participant comparing himself to other PLWHIV, noted that his health status was better. He attributed his good health to his willingness to adhere to treatment in order to minimise the risk of dying. Other comments were:

*I want to make it clear that if you have this illness then you are not part of the living you are as good as dead*, *but with the medication you have a hope of living and therefore you must take it unless you want to die*. (FGD 1)

A focus group participant mentioned that HIV seemed like a death sentence; but with the medication, the hope of survival was better. Another participant mentioned that people who worry about their condition tend to miss their medication. Addressing the fear of death was seen as a positive step to improving adherence and avoiding the consequences. The desire to live and experience good health motivated adherence to ART among participants.

#### Pill mobility enhances adherence

The medication times and schedules of participants varied. However, participants mentioned alternative methods of adhering to treatment despite the disruption of their medication regimen by other activities. Carrying pills when travelling or going out was an example of such an alternative. One participant noted:

*I always make it a point that it is always on me*. *Wherever I am going it’s in my purse*. *At least it’s either you just cut a piece of it*, *you wrap it somewhere and put it in your bag so that you take* …*I think it is working but they should bring something that we don’t have to take or carry all the time so you don’t forget maybe an injection or something else*. (P1)

Participants of focus group discussion three (3) identified that going out without their medication on their person contributed to delaying the medication administration. However, participants also suggested that pill mobility came with a risk of being noticed in public with the ARVs. Nevertheless, a participant mentioned that she sometimes takes her ARVs on a bus while travelling.

#### Status disclosure (Transparency and openness)

Participants’ responses suggested that adherence to treatment required transparency about one’s HIV status, since it enabled PLWHIV to receive support from family members and significant others. However, only a few participants mentioned they could take their medication if others were looking at them.

…*recently I had to inform my younger brother because when I went to his room his wife was pregnant and I saw some of my drug on the table in their room*. *I called him aside and cautioned him about the way he had left the drug ‘we don’t put it like that on a table’ because if someone who is taking it sees it he or she would know (referring to knowing HIV status)*. *So he asked me; do you know what it is*? *I told him that if I did not know I would not say so*. *I took him to my room and showed my medication to him before he believed me*. (P6)

The participant’s transparency about her HIV status and ARV created the opportunity for her brother to disclose his wife’s HIV positive status and active ART. His wife was willing to have her status disclosed to close relations. Transparency about their status demonstrated that they had overcome the barrier of stigma to reduce their risk of non-adherence.

#### Voice calls promote adherence

Evaluating the experiences with alarm use, text messages, and voice calls, as adherence support measures revealed mixed feelings among participants. There was generally a preference for voice calls as the calls were made in an indigenous language, had a greater personal and emotional component, and were also more interactive than text messages. Some participants noted that the calls they received at month three (3) and month six (6) reminding them to turn up for appointments were useful. They advocated that the intervention should be included in the care strategy because they had problems remembering appointment dates. Some of the comments were:

*I really like the calls that coincided with the clinic days because I get to be reminded about my clinic appointment*. (FDG 2)

*You also encouraged us to use the alarm on the phone to remind us take our medication*. *Sometimes I received messages and calls as well*. *I feel that the phone call is good*. (FDG 2)

*The way you called us to come to the clinic is the one I like especially when we finish you give us transport that one is very good*. (P5)

One participant noted that voice calls offered good opportunities for reinforcing adherence counselling and motivating retention in treatment.

### Adherence behaviour inhibitors

Although most of the participants indicated they were adherent to treatment, some mentioned instances of nonadherence. They suggested that adherence is inhibited by situations such as forgetting pills, religious beliefs, oversleeping, secrecy, and wasting time. The evaluation of the mobile phone intervention suggested that text messages were not preferred because of the associated risk of HIV status exposure.

#### Forgetfulness

Some participants reported forgetting to take their medication while others admitted forgetting their appointment schedules.

*I think the doctors themselves know that some people could forget the follow up date*. *I sometimes miss the date*, *but once I notice my medication is reducing*, *I go back to the clinic for refill*. *You have to keep tracking the clinic days otherwise the date would elapse before you realize it*. *If you do not exceed one week then you could still have medications*, *because they always make sure that you receive medication in excess of your reporting day*. (P2)

In spite of the risk of forgetfulness, a focus group discussion one (1) participant mentioned that the mobile phone intervention helped to keep track of time to avoid missing a dose. Making reference to what could account for forgetfulness and missing pills among PLWHIV on ARVs, some participants suggested that worrying about the illness was a key factor.

*I may forget*, *so the phone has to be with me always so that I can check the time*. (FGD 1)

*It is when you are worried about the illness and see it as a problem that you are likely to forget*. (P3)

#### Religious beliefs

Some participants indicated that, although taking the medication was important, some people may be non-adherent to their treatment because of instructions from religious leaders, such as pastors or priests. They gave accounts of other PLWHIV dying because they discontinued taking their medication and pursued religious activities, such as going to prayer camps for prayers, fasting, and deliverance sessions—a form of religious practice considered as an exorcism of evil disease-causing spirits.

*Sometimes some of them abandoned their medication claiming that their pastors have asked them to come to the prayer camp for fasting… Someone was asked by the pastor to come for fasting and deliverance and she has lost her life through that*. (P3)

*Another bad thing is that most people living with HIV listen to their pastors when they visit prayer camps*, *who advise them not to take the drugs*. *They therefore expect to be healed from the prayers which eventually make them weaker and weaker after refraining from taking the medications*. *God is indeed able*, *but stopping the medicine is inexcusable in this regard*. (P2)

Some participants also expressed concerns about seeking religious support without adhering to the ARVs. They clarified that they adhered to the ARVs even while seeking a cure for HIV through religion. They argued that priests who convince PLWHIV to stop treatment were also contributing to the problem of non-adherence. Beyond patient education on adherence, participants did not state what else could be done to curtail the priests’ practice.

#### Nondisclosure of status (Secrecy)

Some participants reported physically hiding to take their medication, in order to avoid stigma. This was because they had not disclosed their HIV status to anyone. Participants suggested that keeping the HIV status secrete spared them from negative comments and stares. Participants who were parents did not disclose their HIV status to their children especially if their children were of a young age (adolescents and younger). A strategy commonly used by some of the participants was changing the pill bottles to disguise their medication.

*I have poured my medicine in a vitamin bottle*, *so in case I am taking it you would not know which medicine I am taking…If you are taking medicine every day for people to see you would subject yourself to unnecessary questions*. *So it is better to keep everything to yourself and hide before taking it*. *You don’t have to expose your medicine anyhow*. (FGD 3)

Participants did not indicate that hiding to take medication prevented them from taking the medication. Nevertheless, the secrecy associated with HIV and its treatment has the potential to affect adherence behaviour. When significant others are aware of the PLWHIV’s status, they might provide support with reminders to take treatment, accompany the PLWHIV to hospital for appointments and provide emotional support.

#### Waiting time

Some of the participants were concerned about the waiting time at the hospital during clinic visits for medication refills. They argued that long waiting hours at the clinic and the pharmacy could discourage them from returning for follow-up appointments.

*Sometimes when you come to the clinic you spend the whole day here waiting*. *From this place you have to go and wait at the pharmacy as well*. (P5)

Some of the participants admitted they were offered preferential treatment when they fabricated excuses as to why they should be served earlier. Still, they were uncomfortable because of how the other people seeking medical attention may have felt each time they were permitted to jump the queue.

#### Sleeping

Two (2) of the participants pointed out in their narratives that they sometimes overslept and, as a result, missed the exact time their next medication dose was due. Participants in the focus groups agreed that sleep sometimes delayed their medication, noting that:

*Well*, *I wasn’t taking it religiously because sometimes I oversleep and my time will pass and if that happens it affects me*. (P1)

The introduction of the mobile phone alarm was beneficial in addressing the problem of sleep. Participants mentioned that they kept the phone close to them so that the alarm would wake them up to take their medication when they overslept.

## Discussion and conclusion

Patients suggested that when the medication regimen was simple, pill burden was minimal and dosing time was convenient, which promoted adherence. These findings are consistent with other studies [6–8,18] on factors influencing adherence to treatment. Generally, motivation to adhere was linked to the benefits of ART and the consequences of non-adherence. The primary benefit of treatment was in the improvement in health status as reported by Portelli et al. [11]. Starks et al. [9] identified that the desire to live motivated adherence; hence, participants were willing to take measures that facilitated adherence, including carrying their pills on their person. However, there were deliberate efforts that ensured the pills were disguised to avoid suspicion and stigma [9,11,18].

The contrast between the use of secrecy in dealing with perceived stigma and the role of openness in promoting ART adherence requires further investigation. We need to understand and address how transparency about HIV status promotes support from spouses, family, and friends, as well as measures for improving HIV status disclosure. There is evidence that affirms that adherence is promoted when HIV status is disclosed because PLWHIV can receive support.

Food was considered an energy buffer that addressed medication side effects, such as body weakness and dizziness. It is, therefore, an important aspect of treatment adherence [19]. Food insecurity is noted as one of the inhibitors affecting treatment adherence. Accordingly, nutritional support has been used as an adherence intervention; and the WHO [1] has recommended supporting the treatment of PLWHIV on treatment with food provisions.

Adherence inhibitors, such as forgetting medication times and sleeping through doses, could be resolved by using mobile phone interventions (alarms). Other studies support how measures such as alarms and text messages have addressed non-adherence [20,21]. However, certain religious beliefs promote non-adherence. For example, the belief that religious practices like prayers and deliverance (exorcism) could cure HIV encourages PLWHIV to seek solutions from religious leaders. Future interventions need to target religious leaders in order to build their knowledge on HIV/AIDS, its causes and evidence-based treatment options. This may promote early referral of PLWHIV to appropriate health centres for medical attention prior to or while religious solutions are sought.

Theoretical interpretation of study outcome from the perspectives of behavioural learning suggests that there are internal and external antecedents explaining adherence behaviour and consequences [22]. The findings revealed personal determination (internal antecedent) precipitated adherence, while the use of the mobile phone (external antecedent) moderated behaviour. The consequence of adhering to the treatment was the benefit derived from the treatment. In understanding these factors, measures for improving adherence promoters and delimiting adherence inhibitors are key in sustaining adherence behaviour.

An elaborate context of describing adherence behaviour in this study is the application of the theory of reasoned action. The theory postulates that behaviour is controlled by individuals’ intention, which predicts behaviour [23]. The intention to adhere depends on behavioural beliefs, perceived norms, and control. Participants’ attitude towards adherence was informed by the belief that making a personal decision to take the medication was important. Participants acknowledged the benefit of the treatment and how it improved their health; hence, their willingness to take their medication. Nevertheless, they were cognizant of the possibility of missing pill times; therefore, they embraced other measures such as alarms, text messages, and voice calls to achieve the desired outcome.

Healthcare professionals working with PLWHIV need to recognise that this population has high levels of motivation to adhere to treatment. Facilitating the development of adherence goals, supporting patients with regular counselling, and providing follow-up care are practical measures that can further promote treatment adherence. There is a need to explore other mechanisms that will reduce clinic waiting time, such as appointment scheduling. Appointment scheduling should be for specific dates and times, in order to reduce waiting times in the health care facilities.

Generally, qualitative studies rely on data saturation in determining the sample size. Nevertheless, additional interviews could have provided more depth for some of the key issues explored, including the influence of religious belief, status disclosure, and access to food on adherence behaviour. The integration of interviews and focus group discussions in presenting the findings may be critiqued by other qualitative researchers as unsuitable. In this study, trained PLWHIV volunteered as interviewers and were supervised by the principal investigator (GD) to conduct some interviews. Their involvement in data collection may have been associated with a risk of interviewer bias. However, the volunteers’ familiarity with the participants appeared to have contributed to participants’ open discussion of the issues related to the promoters and inhibitors of treatment adherence.

## Supporting information

S1 File Interview guides. Interview guides used in study.
(DOCX)

S2 File COREQ checklist. COREQ Checklist used in study.
(PDF)
