# Supplementary material for: Promoters and inhibitors of treatment adherence among HIV/AIDS patients receiving antiretroviral therapy in Ghana: Narratives from an underserved population
Source: PLoS One. 2020 Mar 6;15(3):e0230159. doi: 10.1371/journal.pone.0230159 (PMC7059913; doi:10.1371/journal.pone.0230159)
Supplement: S1 File — Interview guides used in study. (DOCX) [file pone.0230159.s001.docx]

# Appendix D : Interview guide

We had some discussion a few months ago and I informed you we will have a conversation some time later about the taking of your medications. You are free to answer only questions you are comfortable with and I will discontinue the conversation anytime you request. I will record our conversation to enable me remember what we discussed but your name will not be mentioned or written anywhere.

Suggested questions with probes

1. I know we did meet previously but I want to know you more so please tell me about yourself.
   1. I will like to know about you and your family
   2. How long have you been ill?
   3. What work are you doing now
   4. How has the illness affected you
2. One of the important things you are expected to do every day is taking your medication. Tell me more about it.
   1. How were you taking your medication before we met?
   2. What are some of the medication you are taking?
   3. How were you asked to take them?
   4. What are some of the thing you do to help you take your medication as you were told?
   5. What are some of the problems you have taking your medication?
   6. Some people get sick when they start their medication and sometimes the medication has to be changed. Please tell me about your experience.
   7. Who are some of the people who have been of help to you in taking your medicine?
3. Some month ago we started sending you messages and calling you. Please tell me what you think about this experience.
   1. How often were you receiving the calls and messages
   2. What are some of the things about the calling and texting that you are happy about?
   3. Please tell me some of the things you were not happy about that you want addressed.
   4. What do you think about using the alarms?
   5. What are some of the things you want to be done if we have to continue calling you or sending messages?
4. Tell me any other thing you wish to share about your medication taking that we have not talked about.
   1. Some people do not want other people to know they are taking medicine, please what is the case for you?
   2. If you had the opportunity to talk to someone taking the medicine as you are what will you say?

# Appendix E : Focus group discussion guide

We have been interacting for the past sixth month. Some of you were interviewed individually and we mentioned we would have discussion with other collegues present. Kindly note that, you are not expected to mention your name during our conversation. You are free to ask and respond to the questions or decline from participating.

1. Kindly say something briefly about yourself.

Probes

- 1. *What about you and your family?*
  2. *How long have you been ill?*
  3. *What work are you doing now*
  4. *How has the illness affected you and your family*

1. Who would share some of the important things about taking your medication?

Probes

- 1. *How were you taking your medication before we met?*
  2. *What are some of the medication you are taking?*
  3. *How were you asked to take them?*
  4. *What are some of the thing you do to help you take your medication as you were told?*
  5. *What are some of the problems you have taking your medication?*
  6. *Some people get sick when they start their medication and sometimes the medication has to be changed.*
  7. Who are some of the people who have been of help to you in taking your medicine?

1. Who else has additional experience to share? (same probes)
2. Some month ago we started sending you messages and calling you. Please share your experience with us.
   1. How often were you receiving the calls and messages
   2. What are some of the things about the calling and texting that you are happy about?
   3. What were you not happy about that you want addressed.
   4. What do you think about using the alarms?
   5. What are some of the things you want to be done if we have to continue calling you or sending messages?
3. Share any other experiences or disagreement about the medication taking issues raised.
   1. Some people do not want other people to know they are taking medicine, please what is the case for you?
   2. If you had the opportunity to talk to someone taking the medicine as you are what will you say?
